# A resveratrol derivative modulates TRH and TRH‐like peptide expression throughout the brain and peripheral tissues of male rats

**DOI:** 10.1002/edm2.356

**Published:** 2022-07-25

**Authors:** Albert Eugene Pekary, Albert Sattin

**Affiliations:** ^1^ Research VA Greater Los Angeles Healthcare System Los Angeles California USA; ^2^ Center for Ulcer Research and Education VA Greater Los Angeles Healthcare System Los Angeles California USA; ^3^ Department of Medicine University of California Los Angeles California USA; ^4^ Psychiatry Services VA Greater Los Angeles Healthcare System Los Angeles California USA; ^5^ Department of Psychiatry & Biobehavioral Sciences University of California Los Angeles California USA; ^6^ Brain Research Institute University of California Los Angeles California USA

**Keywords:** cerebellum, epididymis, hypothalamus, piriform cortex, prostate

## Abstract

**Introduction:**

Resveratrol and related polyphenols have therapeutic effects ranging from treatment of depression, Alzheimer's and Parkinson's disease, obesity, diabetes, neurodegeneration and ageing. TRH and TRH‐like peptides, with the structure pGlu‐X‐Pro‐NH_2_, where ‘X can be any amino acid reside, have reproductive, caloric‐restriction‐like, anti‐ageing, pancreatic‐β cell‐enhancing, cardiovascular and neuroprotective effects. We hypothesize that TRH and TRH‐like peptides are mediators of the therapeutic actions of the resveratrol derivative pterostilbene (PT).

**Methods:**

Sixteen young adult male Sprague–Dawley rats were divided into four groups. Control group remained on ad libitum chow and water for 10 days. Acute group received ad libitum chow and water for 9 days and then 0.9 g PT/250 g rat chow for 24 h. Chronic animals received PT in chow for 10 days. Withdrawal rats received PT chow for 8 days and then normal chow for 2 days. TRH and TRH‐like peptide levels were measured in medulla oblongata (MED), frontal cortex (FCX), hypothalamus (HY), amygdala (AY), hippocampus (HC), piriform cortex (PIR), nucleus accumbens (NA), entorhinal cortex (ENT), striatum (STR), cerebellum (CBL), anterior cingulate (ACNG), posterior cingulate (PCNG), prostate (PR), liver (L), testis (T), heart (H), pancreas (PAN), adrenals (AD) and epididymis (EP).

**Results:**

Significant changes in the levels of TRH and TRH‐like peptides occurred throughout the brain and peripheral tissues in response to PT treatment.

**Conclusion:**

The high responsiveness of PIR, CBL, HY, STR, PCNG, MED, FCX, NA, ACNG and AY in brain and EP and PR is consistent with TRH and TRH‐like peptides participating in the therapeutic effects of PT.

## Graphical abstract

Resveratrol and related polyphenols have therapeutic effects ranging from treatment of depression, Alzheimer's and Parkinson's disease, obesity, diabetes, neurodegeneration and aging. TRH and TRH‐like peptides, with the structure pGlu‐X‐Pro‐HN2, where "X" can be any amino acid residue, have reproductive, caloric‐restriction‐like, anti‐aging, pancreatic‐beta cell‐enhancing, cardiovascular, and neuroprotective effects. Our hypothesis: TRH and TRH‐like peptides are mediators of the therapeutic actions of the resveratrol derivative pterostilbene.

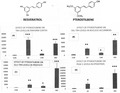

## 1 INTRODUCTION

Resveratrol and related polyphenols such as pterostilbene (PT) have therapeutic effects ranging from treatment of depression and anxiety,^1^, ^2^, ^3^, ^4^, ^5^ stress,^6^, ^7^, ^8^ PTSD,^5^ epilepsy,^9^, ^10^ Alzheimer's and Parkinson's disease,^11^, ^12^, ^13^ diabetes,^13^, ^14^ obesity,^15^ cancer,^16^, ^17^, ^18^ traumatic brain injury,^5^ Huntington's disease,^19^ hypertension,^20^ pain,^5^, ^21^ neurodegeneration^22^, ^23^, ^24^, ^25^ and ageing.^19^, ^26^, ^27^, ^28^, ^29^, ^30^ Resveratrol upregulates mitochondria‐located antioxidant enzymes and triggers mitochondrial biogenesis.^31^

TRH and TRH‐like peptides, with the structure pGlu‐X‐Pro‐NH_2_, where ‘X can be any amino acid reside, have antidepressant, anti‐epileptic, analeptic, reproductive, caloric‐restriction‐like, anti‐ageing, pancreatic‐β cell‐enhancing, cardiovascular and neuroprotective effects.^32^ The TRH/TRH‐R1 receptor signalling pathway is an important mediator of brain–gut axis communication via the brain medulla.^33^ TRH and TRH‐like peptides occur not only throughout the CNS but also peripheral tissues, with particularly high levels in rat and human prostate.^32^, ^34^ Resveratrol decreases both serum TSH and hypothalamic TRH mRNA expression in sub‐clinically hypothyroid rats.^2^

Resveratrol promotes expression of sirtuins (SIRTs).^28^, ^33^ SIRTs are a family of NAD^+^‐dependent enzymes that catalyse post‐translational modifications of proteins. They regulate cellular functions and are associated with ageing and longevity. Dysregulation of SIRTs plays an important role in major diseases, including cancer and metabolic, cardiac and neurodegenerative diseases.^35^

Sulphated metabolites accumulate in the gut following oral ingestion of resveratrol where they promote the growth of beneficial bacteria such as Lactobacillus reuteri and up‐regulate the expression of tight junction and mucin‐related proteins.^36^ Perturbation of the gut microbiome by Rifaximin, an antibiotic which does not cross the gut–blood barrier, has a profound effect on the expression of reproductive and brain TRH and TRH‐like peptides.^34^

**FIGURE 1:**
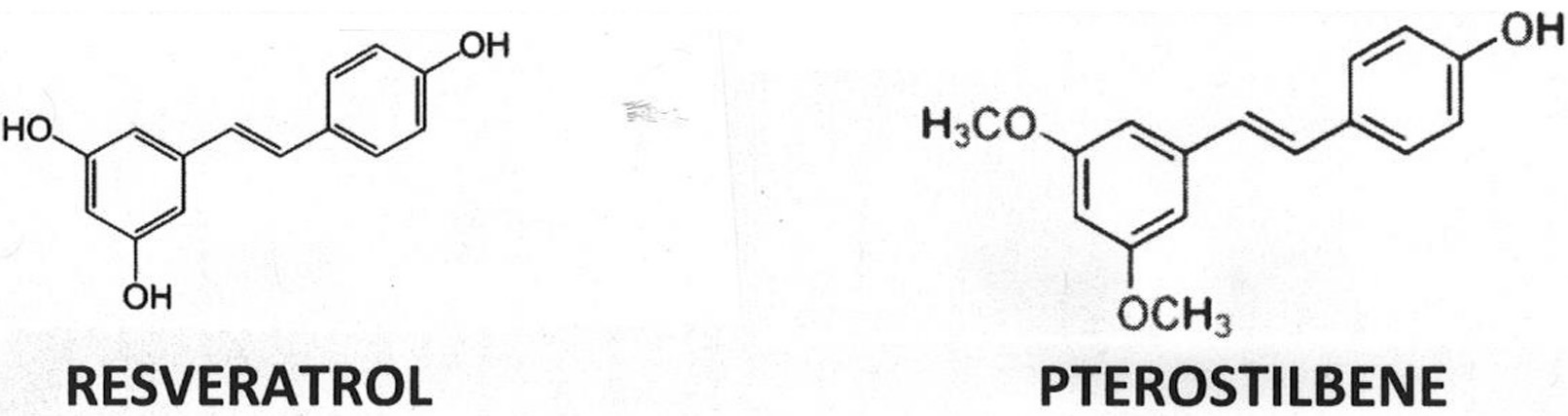
Pterostilbene (PT) is a resveratrol derivative containing two added methyl groups which increase it's lipophilicity and bioavailability

The present studies investigate the potential of TRH and TRH‐like peptides being downstream mediators of PT because: (1) this polyphenol (see Figure 1) readily crosses the blood–brain barrier resulting in increased bioavailability, clearance time and therapeutic potential compared to resveratrol^37^, ^38^ and (2) TRH and TRH‐like peptides are important mediators of intracellular functions, which overlap those of PT and resveratrol, but are rapidly degraded by blood enzymes and cannot cross blood–tissue barriers.^32^

## 2 EXPERIMENTAL PROCEDURES

### 2.1 Animals

‘Young adult male Sprague‐Dawley rats (*n* = 16, SPF, Envigo) were used for all experiments. These animals were group housed (2 animals per cage) on wood shavings with a red plastic tube for play and shelter. Standard Purina rodent chow #5001 and water were provided ad libitum during a standard one‐week initial quarantine with 22 ± 2°C and 50 ± 10% relative humidity; lights on: 6 am to 6 pm. Cages, water and bedding were changed every 3 days. All animals were weighed on the day of receipt and on the morning of each experiment. Initial body weights did not differ between experimental groups. Animals were randomized prior to the start of PT treatment. Research was approved by the VA Greater Los Angeles Healthcare System Animal Care and Use Committee (IACUC Protocol #030090‐10) and conducted in compliance with the Animal Welfare Act and the federal statutes and regulations related to animals and experiments involving animals and adheres to principles stated in the Guide for the Care and use of Laboratory Animals, Eighth Edition, NRC Publication, 2011. All efforts have been made to minimize the number of animals used and their suffering. Animal was handled for 10 min per day for one month and then transferred from the Veterinary Medical Unit to the laboratory 12 h before the start of experiments to minimize the stress of a novel environment’.^32^ ‘The American Veterinary Medical Association has concluded that decapitation without prior sedation “is conditionally acceptable if performed correctly, and it should be used in research settings when its use is required by the experimental design and approved by the Institutional Animal Care and Use Committee”’.^39^ This study is reported in accordance with ARRIVE guidelines (Animal Research: Reporting of In Vivo Experiments) (https://arriveguidelines.org).

‘Because of the 10‐ to 100‐fold changes in TRH and TRH‐like peptide levels in response to the estrus cycle. female rats were not included in the present study’.^40^

### 2.2 Effect of acute, chronic and withdrawal treatment with PT in normal rat chow on levels of TRH and TRH‐like peptides in rat brain and peripheral tissues

Sixteen young adult male Sprague–Dawley rats (7 weeks), body weight (mean ± SD) 203 ± 6 g, were divided into four groups (*n* = 4/group). PT chow was prepared by adding 0.9 g PT (Sigma) to 250 g of standard rodent chow and blending thoroughly with a Ninja Model BL610 Professional 1000 W blender for 30 s. The control (CON) group remained on ad libitum standard chow and water for 10 days until decapitation. The acute (AC) group received ad libitum chow and water for 9 days and then PT chow for 24 h. Assuming 25 g chow consumption/day, this would provide 300 mg PT/kg body weight for 300 g rats. The chronic (CHR) animals received PT chow for 10 days. The withdrawal (WD) rats received PT chow for 8 days and then normal chow for 2 days. The effect of PT withdrawal on TRH and TRH‐like peptide levels when compared to the corresponding acute effects can reveal the relative contribution of changes in peptide biosynthesis (hours) to changes in peptide release (minutes).^34^

### 2.3 Dissection of rat brain and peripheral tissues

All rats were decapitated without anaesthesia to avoid rapid, anaesthetic‐induced, blockade of peptide release.^41^ Nucleus accumbens (NA), amygdala (AY), frontal cortex (FCX), cerebellum (CBL), medulla oblongata (MED), anterior cingulate (ACNG), posterior cingulate (PCNG), striatum (STR), piriform cortex (PIR), hippocampus (HC), entorhinal cortex (ENT), adrenals (AD), pancreas (PAN), prostate (PR), epididymis (EP), testis (T), heart (H) and liver (L) were hand dissected, weighed rapidly and then extracted as previously described in detail.^32^

### 2.4 Serum hormone assays

Serum rat leptin, rat insulin, testosterone, free T_4¸_ total T_3_ and glucose were measured (assay range, intra‐assay CV%) with the following commercial RIA kits: rat leptin (0.801–200 ng/ml, 3.2) and rat insulin (0.0329–2.0 ng/ml, 4.8) (Linco Research, Inc.), testosterone (0.05–40 ng/ml, 6.7), free T_4_ (0.045–60 ng/dl, 4.6) and total T_3_ (0.06–80 pg/ml, 4.8) (MP Biomedical). Serum glucose was measured with the Contour Next EZ Blood Glucose Monitoring System (Ascensia Diabetes Care US, Inc.).

### 2.5 HPLC and RIA procedures, HPLC peak identification and quantitation

HPLC and RIA procedures, peak identification, and quantitation by co‐chromatography with synthetic TRH and TRH‐like peptides, relative potency analysis of multiple antibodies to TRH and TRH‐like peptides, and mass spectrometry for comparing peak areas have been previously reported in detail.^32^, ^33^, ^34^, ^35^, ^36^, ^37^, ^38^, ^39^, ^40^, ^41^, ^42^, ^43^, ^44^, ^45^

Briefly, after boiling, tissues were dried, re‐extracted with methanol, dried and defatted by water—ethyl ether partitioning. Dried samples were dissolved in 0.1%trifluroacetic acid (TFA) and loaded onto reverse phaseC18 Sep‐Pak cartridges (Water). TRH and TRH‐like peptides were eluted with 50% methanol. Dried peptides were again dissolved in TFA, filtered and then fractionated by HPLC using a 4.6 mm × 150 mm Econosphere, 3 mm C18 reverse phase column (Dr. Maisch GmbH) and a 0.2%/min gradient of acetonitrile. The 0.5 ml fractions collected were dried completely and reconstituted with 0.10 ml of 0.02% NaN_3_ just before RIA.

The antiserum used (8B9) cross‐reacts with TRH and nine TRH‐like peptides with a relative potency of displacement ranging from 2.31 (Lys‐TRH) to 0.288 (Ser‐TRH) relative to Tyr‐TRH [Table 2, Ref. 42]. Two of the regularly observed peaks (2a and 2b) consist of a mixture of unidentified TRH‐like peptides. Of the eight observed peptides, three have so far been confirmed by mass spectrometry: TRH, Glu‐TRH and Tyr‐TRH.^43^ Tissue samples from the 4 rats within each treatment group were pooled prior to HPLC to provide the minimum amount of immunoreactivity needed for reliable RIA measurements.

The mean recovery of TRH and TRH‐like peptide immunoreactivity from all tissues studied was 84 ± 15% (mean ± SD). The within‐assay and between‐assay coefficient of variation for measuring 333 pg/ml TRH was 4.8% and 16.9%, respectively. All HPLC fractions obtained from a given brain region or peripheral tissue were analysed in the same RIA. The minimum detectable dose for TRH was 5 pg/ml. The specific binding of [^125^I]TRH (Bo/T) was 25%.

### 2.6 Statistical analysis

Statistical methods for comparing peak areas were made with the aid of Statview (Abacus Concepts, Inc.), a statistical software package for the Macintosh computer. All multi‐group comparisons were carried out by one‐way analysis of variance using post hoc Scheffe contrast with the control group.

The mean within‐group coefficient of variation (CV) (SD/mean, CV‐within group) for each tissue and TRH/TRH‐like peptide combination, across four photoperiod intervals, has been previously reported (circadian rhythm experiment) for untreated Sprague–Dawley male rats.^46^ Mean within‐group CVs in brain ranged from 4.5% for TRH levels in AY to 43% for Phe‐TRH in HY, and from 12% for Val‐TRH in testis to 41% for Trp‐TRH in EP for peripheral tissues. These CVs were then used to estimate the level of significance, by one‐way ANOVA, of changes in the pooled mean values (see Ref. [47]) of TRH and TRH‐like peptide levels following acute (AC), chronic (CHR) and withdrawal (WD) ingestion of PT. Pooling of at least 4 tissue extracts was required to provide sufficient signal‐to‐noise in the RIA for many brain regions and to keep the total number of HPLC fractions to be analysed reasonable: 4 treatment groups × 19 tissues × 100 HPLC fractions/tissue pool = 7600 RIA samples for the present study. Without pooling the total number of HPLC fractions would have been 4 × 7600 = 30,400.

## 3 RESULTS

### 3.1 Body weights

Mean body weights for all animals at the time of decapitation were 333.5 ± 20.0 g. Mean animal weights for each PT treatment group did not differ significantly with the untreated controls by one‐way ANOVA.

### 3.2 Serum hormone levels following oral PT

Serum glucose levels for the CHR group were significantly lower than the CON group (*p* < .05). All other serum hormone levels did not differ significantly between corresponding experimental groups by one‐way ANOVA (Table 1).

**TABLE 1:** Effect of oral pterostilbene on serum hormone levels of male rats

	Testosterone Nmol/L	fT_3_ pg/ml	fT_4_ ng/dl	Leptin ng/ml	Rat insulin ng/ml	Glucose mg/dl	CORT ng/ml
CON	10.0 ± 4.2	2.61 ± 0.30	2.49 ± 0.42	1.40 ± 0.25	0.15 ± 0.04	97 ± 10	139 ± 32
AC	11.5 ± 3.1	1.90 ± 0.35	2.02 ± 0.17	1.86 ± 0.51	0.15 ± 0.01	109 ± 11	301 ± 35
CHR	10.1 ± 7.2	2.43 ± 0.45	2.43 ± 0.43	1.79 ± 0.27	0.15 ± 0.01	97 ± 6*	238 ± 35
WD	12.3 ± 7.1	2.54 ± 0.46	2.51 ± 0.49	1.67 ± 0.16	0.15 ± 0.02	120 ± 5	243 ± 102

*Note:* All values are mean ± SD.

Abbreviations: AC, acute; CHR, chronic; CON, control; WD, withdrawal.

*p < .05 by one‐way ANOVA using post hoc Scheffe contrasts with control rats.

### 3.3 Overview of TRH and TRH‐like peptide data

Our combined HPLC‐RIA methodology can resolve 10 TRH and TRH‐like peptides: Glu‐TRH, Peaks 2a and 2b (partially resolved mixture of TRH‐like peptides), TRH, Val‐TRH, Thr‐TRH, Tyr‐TRH, Leu‐TRH, Phe‐TRH and Trp‐TRH.^48^ The present study evaluated 12 brain regions and 7 peripheral tissues for the PT experiment. This represents 10 × 19 = 190 peptide mean values.

**FIGURE 2:**
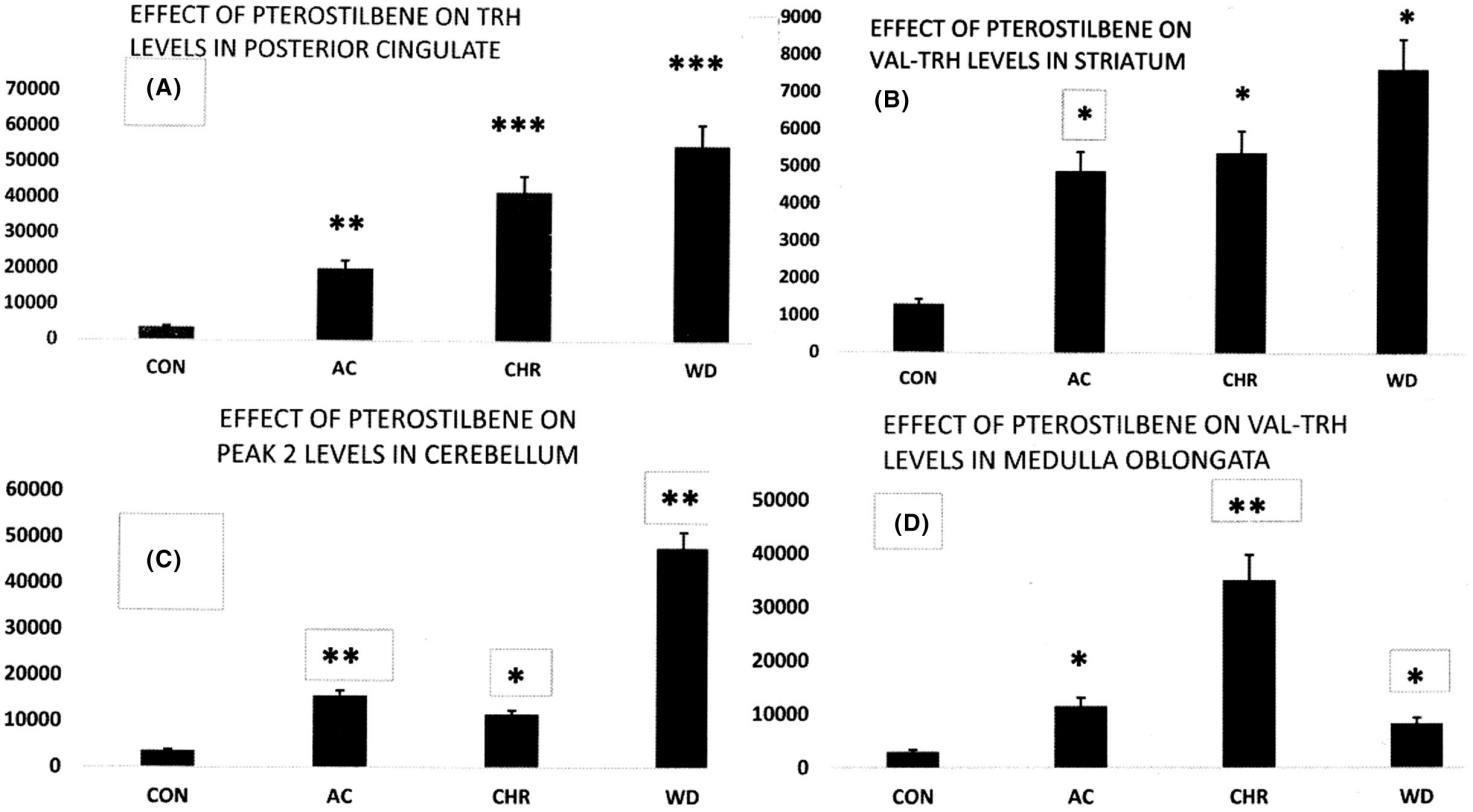
Representative profiles of TRH and TRH‐like peptide responses in male rats to pterostilbene (PT) treatment. The response profiles in (A–C) could be explained by PT‐induced peptide biosynthesis along with a compensatory increase in peptide release. Withdrawal of PT results in a rapid decrease in peptide release (further increase in peptide level) while onset of decline in PT‐induced biosynthesis is much slower. The profile in (D) suggests PT‐induced peptide release, which is compensated by increased PT‐dependent peptide biosynthesis. Upon PT withdrawal, synthesis declines but increased release persists. *p < 0.05; **p < 0.01; ***p < 0.002

**FIGURE 3:**
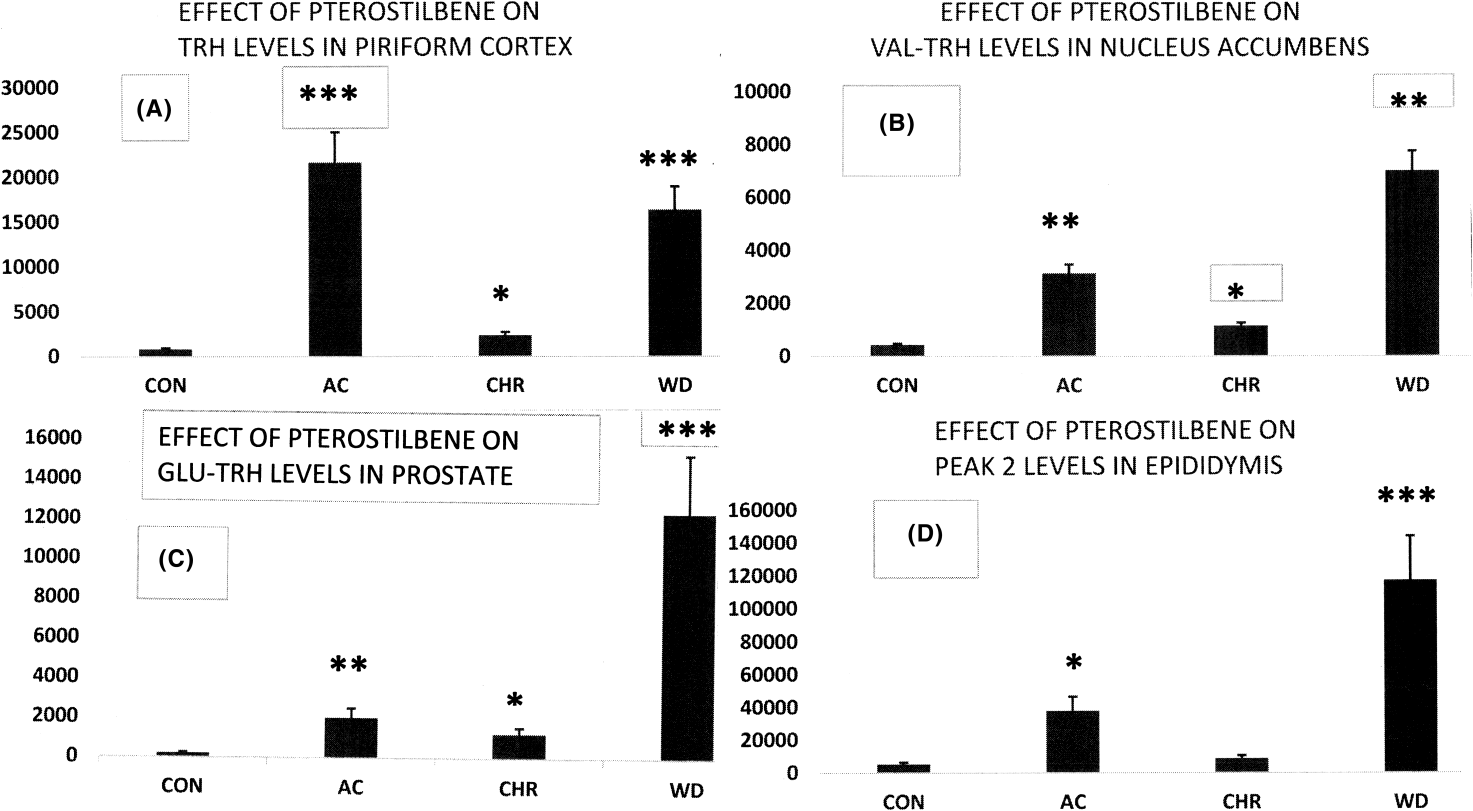
Representative profiles of TRH and TRH‐like peptide responses in male rats to pterostilbene (PT) treatments. The response patterns in (A–D) are consistent with PT‐stimulation of rapid‐onset but a slowly deceasing rate of PT‐dependent biosynthesis which is compensated by a slow‐onset increase in peptide release. Upon PT withdrawal, peptide release stops abruptly (increased peptide level) while PT‐induced biosynthesis declines much more slowly. *p < 0.05; **p < 0.01; ***p < 0.002

The number of significant changes in TRH and TRH‐like peptide levels in brain resulting from PT treatment (in parentheses), in descending order were as follows: PIR(16), CBL(16), HY(15), STR(14), PCNG(12), MED(11), NA(10), ACNG(10), AY(9) and FCX(7) as seen in Table 2 and Figures 2 and 3. The corresponding ranking for peripheral tissues were as follows: EP(17), PR(13), AD(8), H(5), T(4), L(3) and PAN(2) (See Table 3 and Figure 3).

**TABLE 2:** Effect of oral pterostilbene on TRH and TRH‐like peptide levels in brain regions of male rats (pg)

	Glu‐TRH	Peak 2	TRH	Val‐TRH	Tyr‐TRH	Leu‐TRH	Phe‐TRH	Trp‐TRH
Hypothalamus								
CON	1641 ± 542	21,898 ± 7883	27,404 ± 8769	1727 ± 535	832 ± 216	1180 ± 484	435 ± 187	726 ± 203
AC	1423 ± 470	6307 ± 2271*	59,590 ± 19,069*	28,062 ± 8699**	3063 ± 796*	6751 ± 2768*	2845 ± 1223*	727 ± 204
CHR	333 ± 110**	2339 ± 842**	162,406 ± 51,970*	1790 ± 555	1430 ± 372	1197 ± 491	703 ± 302	374 ± 105
WD	7952 ± 2624**	19,431 ± 6995	50,078 ± 16,025	4246 ± 1316*	1803 ± 469*	30,851 ± 12,649***	4525 ± 1946**	13,094 ± 3666**
Amygdala								
CON	1144 ± 324	2878 ± 386	11,732 ± 528	2592 ± 246	1364 ± 232	1836 ± 494	1634 ± 255	1686 ± 357
AC	1142 ± 323	3039 ± 407	19,854 ± 893	1185 ± 113*	1831 ± 311	1822 ± 490	1373 ± 214	1305 ± 277
CHR	1825 ± 516	2688 ± 360	26,207 ± 1179*	1067 ± 101*	2087 ± 355	949 ± 255	1293 ± 202	3444 ± 730*
WD	2461 ± 696*	13,951 ± 1869**	22,514 ± 1013	2002 ± 190	2091 ± 355	2158 ± 581	4679 ± 730*	4406 ± 934*
Piriform cortex								
CON	515 ± 102	1556 ± 230	804 ± 125	2817 ± 397	248 ± 51	372 ± 81	769 ± 158	227 ± 72
AC	2872 ± 569**	11,505 ± 1703**	21,613 ± 3372***	3557 ± 502	0	2417 ± 529**	2902 ± 595*	633 ± 202*
CHR	296 ± 59	1445 ± 214	2358 ± 368*	119 ± 17***	203 ± 42	424 ± 93	745 ± 153	274 ± 87
WD	2300 ± 455*	26,206 ± 3878**	16,312 ± 2545***	1298 ± 183*	645 ± 132*	7213 ± 1580***	6571 ± 1347**	3949 ± 1256**
Nucleus accumbens								
CON	544 ± 58	1423 ± 115	6711 ± 852	433 ± 55	887 ± 100	473 ± 134	577 ± 65	299 ± 57
AC	766 ± 81	2056 ± 167	3694 ± 469	3138 ± 399**	525 ± 59	701 ± 198	601 ± 68	327 ± 62
CHR	534 ± 57	2924 ± 237*	8858 ± 1125	1146 ± 146*	388 ± 44*	445 ± 126	558 ± 63	456 ± 87
WD	1609 ± 171*	4616 ± 374*	6000 ± 762	7002 ± 889***	3304 ± 373*	764 ± 216	1978 ± 224*	1208 ± 231**
Striatum								
CON	3122 ± 375	23,845 ± 3529	68,139 ± 6746	1309 ± 204	1310 ± 204	1350 ± 162	1029 ± 50	1007 ± 107
AC	5888 ± 707	17,100 ± 2531	38,066 ± 3769	4888 ± 763*	4168 ± 650*	3160 ± 379*	2584 ± 127*	3202 ± 339*
CHR	4674 ± 561	7000 ± 1036*	47,714 ± 4724	5398 ± 842*	2518 ± 393	3486 ± 418*	1516 ± 74	2480 ± 263*
WD	3392 ± 407	64,982 ± 9617*	35,520 ± 3516	7642 ± 1192*	2168 ± 338	2168 ± 260	2520 ± 123*	2716 ± 288*
Medulla oblongata								
CON	5730 ± 974	25,344 ± 2332	8537 ± 1024	2998 ± 402	3662 ± 725	2331 ± 611	1200 ± 246	1620 ± 309
AC	4631 ± 787	29,077 ± 2675	34,156 ± 4099**	11,562 ± 1549*	6002 ± 1188	3954 ± 1036	2923 ± 599*	1831 ± 350
CHR	4878 ± 829	10,688 ± 983	11,391 ± 1367	35,069 ± 4699**	3088 ± 611	5683 ± 1489*	2347 ± 481	2812 ± 537
WD	9090 ± 1545	47,888 ± 4406	39,604 ± 4752**	8240 ± 1104*	10,220 ± 2024*	5607 ± 1469*	7344 ± 1506**	4414 ± 843*
Cerebellum								
CON	1264 ± 99	3646 ± 259	10,038 ± 1345	4715 ± 500	2357 ± 332	5004 ± 1626	1670 ± 189	1276 ± 361
AC	3309 ± 258*	15,612 ± 1108**	9059 ± 1214	9574 ± 1015*	9355 ± 1319*	2761 ± 897	4293 ± 485*	2282 ± 646
CHR	2807 ± 219*	11,569 ± 821*	10,478 ± 1404	11,449 ± 1214*	2286 ± 322	4477 ± 1455	3465 ± 392*	2034 ± 576
WD	4945 ± 386*	47,624 ± 3381**	27,482 ± 3683*	8040 ± 852	12,811 ± 1806**	13,275 ± 4314*	8475 ± 958**	12,313 ± 3485***
Anterior cingulate								
CON	997 ± 99	3268 ± 438	2494 ± 546	1001 ± 198	476 ± 74	1409 ± 478	896 ± 228	579 ± 115
AC	959 ± 95	1538 ± 206*	3512 ± 769	791 ± 157	441 ± 69	908 ± 308	1051 ± 268	507 ± 100
CHR	225 ± 22**	1404 ± 188*	3203 ± 701	262 ± 52*	524 ± 82	213 ± 72**	461 ± 118	312 ± 62
WD	1473 ± 146	3797 ± 509	11,383 ± 2493**	319 ± 63*	2074 ± 324**	225 ± 76**	915 ± 233	1778 ± 352*
Posterior cingulate								
CON	1664 ± 341	3796 ± 725	3671 ± 389	3267 ± 670	886 ± 144	3019 ± 1132	1924 ± 327	1538 ± 337
AC	3527 ± 723*	4552 ± 869	20,366 ± 2159**	619 ± 127**	2744 ± 447*	1300 ± 488*	1507 ± 256	1073 ± 235
CHR	2280 ± 467	518 ± 99**	41,896 ± 4441***	1990 ± 408	1175 ± 192	1795 ± 673	3011 ± 512	1427 ± 313
WD	2743 ± 562	20,990 ± 4009**	55,033 ± 5833***	36,165 ± 7414***	2041 ± 333*	3017 ± 1131	5987 ± 1018*	1950 ± 427
Frontal cortex								
CON^a^	—	—	—	—	—	—	—	—
AC	4055 ± 487	16,997 ± 4334	8691 ± 1721	6410 ± 949	1160 ± 74	3744 ± 528	6492 ± 824	1344 ± 176
CHR	3325 ± 399	6155 ± 1569	34,929 ± 6916	2089 ± 309	1356 ± 87	1971 ± 278	4526 ± 575	2687 ± 352
WD	11,012 ± 1321^b^	34,751 ± 8862	100,922 ± 19,983^b^	120,801 ± 17,879^c^	10,652 ± 682^c^	50,476 ± 7117^c^	484,100 ± 61,481^c^	13,050 ± 1710^c^

*Note:* ^a^CON group for pooled FCX lost during extraction process. ^b^ *p* < .01, ^c^ *p* < .001 versus the AC group. All values are mean ± SD.

*p < .05; **p < .01 and ***p < .002 by one‐way ANOVA using post hoc Scheffe contrasts versus the CON group.

Abbreviations: AC, acute; CHR, chronic; CON, control; FCX, frontal cortex; WD, withdrawal.

**TABLE 3:** Effect of oral pterostilbene on TRH and TRH‐like peptide levels in peripheral tissues of male rats (pg)

	Glu‐TRH‐	Peak 2	TRH	Val‐TRH	Tyr‐TRH	Leu‐TRH	Phe‐TRH	Trp‐TRH
Adrenals								
CON	1202 ± 385	4739 ± 711	1951 ± 390	1214 ± 352	2098 ± 965	2233 ± 737	869 ± 235	949 ± 332
AC	1033 ± 331	4779 ± 717	2854 ± 571	626 ± 182	816 ± 375*	1737 ± 573	2972 ± 802*	1286 ± 437
CHR	785 ± 251	1872 ± 281*	809 ± 162*	1457 ± 423	712 ± 328*	1016 ± 335*	943 ± 255	1621 ± 551
WD	1223 ± 391	5185 ± 778	3779 ± 756	1907 ± 553	4210 ± 1937*	3996 ± 1319	2876 ± 777*	1119 ± 380
Epididymis								
CON	10,138 ± 3143	5175 ± 1708	5736 ± 1434	4389 ± 1448	14,616 ± 4385	6655 ± 1464	14,284 ± 1714	4537 ± 1860
AC	24,905 ± 7721*	37,767 ± 12,463*	13,296 ± 3324*	2749 ± 907	2425 ± 728**	11,414 ± 2511	25,422 ± 3051	27,850 ± 11,419*
CHR	10,764 ± 3337	8645 ± 2853	2804 ± 701*	4157 ± 1372	1500 ± 450**	2232 ± 491*	1955 ± 235**	1702 ± 698*
WD	43,488 ± 13,481**	116,684 ± 38,506***	56,272 ± 14,068**	39,955 ± 13,185**	19,395 ± 5819	90,957 ± 20,011**	49,501 ± 5940*	54,245 ± 22,240**
Prostate								
CON	211 ± 72	8059 ± 2579	259,466 ± 70,056	20,572 ± 5554	87,170 ± 27,023	195,010 ± 72,154	60,608 ± 18,788	72,909 ± 22,602
AC	2018 ± 686**	24,966 ± 7989*	692,873 ± 18,7076*	91,780 ± 24,781*	210,706 ± 65,319*	113,261 ± 41,907	89,028 ± 27,599	56,383 ± 17,479
CHR	1244 ± 423*	63,305 ± 20,258*	94,085 ± 25,403*	15,422 ± 4164	35,845 ± 11,112*	91,463 ± 33,841*	38,344 ± 11,887	18,164 ± 5631
WD	12,379 ± 4209***	40,485 ± 12,955*	461,850 ± 124,700	81,411 ± 21,981*	130,464 ± 40,444	165,815 ± 61,352	82,344 ± 25,527	72,961 ± 22,618
Testis								
CON	572 ± 132	2621 ± 996	3131 ± 658	4745 ± 569	837 ± 243	2716 ± 760	2333 ± 537	383 ± 115
AC	1038 ± 239	5040 ± 1915	4819 ± 1012	11,720 ± 1406*	513 ± 149	4945 ± 1385	3960 ± 911	846 ± 254*
CHR	541 ± 124	2750 ± 1045	2200 ± 462	4405 ± 529	2480 ± 719*	1521 ± 426	2055 ± 473	455 ± 137
WD	288 ± 66*	2909 ± 1109	1753 ± 368	5292 ± 635	1271 ± 369	4114 ± 1152	3206 ± 737	413 ± 124
Pancreas								
CON	1265 ± 443	3450 ± 1173	2312 ± 971	834 ± 192	1174 ± 247	1551 ± 357	819 ± 172	1641 ± 361
AC	945 ± 331	2873 ± 977	1890 ± 794	1468 ± 338	757 ± 159	2203 ± 507	964 ± 203	2078 ± 457
CHR	2117 ± 741	6234 ± 2120	2503 ± 1051	2367 ± 544*	1136 ± 239	1622 ± 373	904 ± 190	1690 ± 372
WD	2097 ± 734	2688 ± 914	1777 ± 746	1335 ± 307	2087 ± 438*	1387 ± 319	1471 ± 309	688 ± 151
Liver								
CON	1655 ± 397	3660 ± 695	3207 ± 1251	828 ± 240	1415 ± 425	674 ± 216	883 ± 283	1267 ± 291
AC	1252 ± 300	3181 ± 604	1064 ± 415*	1021 ± 296	601 ± 180	871 ± 279	1237 ± 396	1321 ± 304
CHR	1325 ± 318	2741 ± 521	789 ± 308*	769 ± 223	655 ± 197	1531 ± 490	3732 ± 1194*	664 ± 153
WD	1466 ± 352	4111 ± 781	1367 ± 533	861 ± 250	1995 ± 599	1081 ± 346	1473 ± 471	1764 ± 406
Heart								
CON	232 ± 49	953 ± 123	1945 ± 175	1000 ± 220	1120 ± 246	1592 ± 350	2999 ± 720	610 ± 153
AC	256 ± 54	448 ± 58	1844 ± 166	831 ± 183	1188 ± 261	2991 ± 658	3454 ± 829	395 ± 99
CHR	554 ± 116	846 ± 110	1638 ± 147	1643 ± 361	2511 ± 552*	3704 ± 815*	5570 ± 1337	617 ± 154
WD	1100 ± 231*	2064 ± 268*	1247 ± 112*	516 ± 114	865 ± 190	647 ± 142	1476 ± 354	1092 ± 273

*Note*: All values are mean ± SD.

*p < .05, **p < .01 and ***p < .002 by one‐way ANOVA comparison with the control group using Scheffe contrasts.

## 4 DISCUSSION

Acute, chronic and withdrawal treatment with PT results in significant increases in TRH, Leu‐TRH, Trp‐TRH, Phe‐TRH, Tyr‐TRH, Glu‐TRH and Peak 2 and decreases in Val‐TRH for piriform cortex (Table 2 and Figure 2). These changes result from alterations in the biosynthesis and/or release of these tripeptides.^34^ These remarkable changes in peptide levels within the piriform cortex are consistent with current knowledge regarding the role of TRH (and TRH‐like peptides) as mediators of antidepressant effects in mammalian brain.^32^ The antidepressant activity of Tyr‐TRH and analeptic effect of Val‐TRH correspond with actions of TRH.^32^ TRH and TRH‐like peptide biosynthesis occurs within large dense core vesicles (LDCV) of glutamatergic neurons. They are co‐released with glutamate and act to moderate the effects of this excitotoxic neurotransmitter.^32^ Neuropeptides, such as TRH, which are co‐released with classical neurotransmitters are now considered primary mediators of brain circuit connectivity with a longer duration of action.^49^

TRH and TRH‐like peptide levels were increased by PT treatments in cerebellum, as seen in Table 2 and Figure 2. Pharmacological activation of SIRT1 with resveratrol significantly reduces motor incoordination of Machado‐Joseph disease mice, a degenerative disorder characterized by cerebellar ataxia.^50^ Caloric restriction blocks this neuropathology and motor deficits.^50^ The anti‐ataxic effects of TRH and analogs have been investigated in rolling mouse Nagoya or 3‐acetylpyridine treated rats, which are regarded as a model of human cerebellar degenerative disease. TRH differentially affects clinical cerebellar ataxia.^51^ TRH participates in cerebellar long‐term depression and motor learning.^52^

The traditional view of the cerebellum is that it controls motor behaviour. The cerebellum also plays an important role in normal and impaired social behaviours such as autism spectrum disorder^53^ as does resveratrol.^54^

Chronic treatment with PT increased the level of hypothalamic TRH, as shown in Table 2, which has antidepressant and anti‐PTSD effects.^32^ Trans‐resveratrol protects neurons against PTSD through regulation of limbic hypothalamus–pituitary–adrenal axis function and activation of neuroprotective molecules such as protein kinase A, phosphorylated cAMP response element‐binding protein and brain‐derived neurotrophic factor expression.^5^ Antidepressant‐like effect of trans‐resveratrol involves the regulation of the central serotonin and noradrenaline levels and related MAO‐A activities.^8^

Treatment with PT increased STR levels of TRH‐like peptides including Tyr‐TRH, Phe‐TRH and Val‐TRH (Table 2 and Figure 2) which have antidepressant effects.^32^ Resveratrol can reverse the dysregulation of mitochondrial respiration in models of Huntington's disease which selectively affects the striatum and cortex.^19^

Significant increases in TRH levels in posterior cingulate accompanied PT treatments (Table 2 and Figure 2). The posterior cingulate shows abnormalities in a range of neurological and psychiatric disorders including Alzheimer's disease, schizophrenia, autism, depression, attention deficit hyperactivity disorder and ageing.^27^ Resveratrol treatment normalizes the peripubertal stress‐induced social investigation deficit.^7^

Microinjection of resveratrol into rostral ventrolateral medulla decreases sympathetic vasomotor tone through nitric oxide and intracellular Ca^2+^ in anaesthetized male rats.^55^ The resulting reduction in blood pressure, heart rate and renal sympathetic nerve activity is consistent with a decreased TRH release rate resulting in increased TRH levels, Table 2, following acute and withdrawal treatment with PT. TRH increases blood pressure, heart rate and renal sympathetic nerve activity.^32^

Chronic social defeat stress, a model of depression in rodents, increases SIRT1 levels in the nucleus accumbens, a key brain reward region. Resveratrol, a pharmacological activator of SIRT1, when infused bilaterally into the NA, increased depression‐ and anxiety‐like behaviours.^56^ Increased levels of TRH in NA following acute, chronic and withdrawal PT treatment (Table 2), is consistent with increased biosynthesis and release of TRH which has antidepressant and anxiolytic actions in male rats.^32^ Anterior cingulate inputs to the nucleus accumbens control the social transfer of pain and analgesia.^57^ The marked PT‐induced changes in TRH and TRH‐like peptide levels (Table 2, Figures 2 and 3) within the cingulate and nucleus accumbens are noteworthy given these important neural and cognitive linkages between these brain regions. SIRT1 in the brain is involved with ageing‐associated disorders and lifespan.^58^

The posteromedial nucleus of the cortical amygdala contains TRH‐expressing neurons that control mating behaviour.^59^ Chronic PT treatment increased TRH levels in the amygdala. (Table 2). Amygdala TRH levels fluctuate significantly during the rat oestrus cycle.^40^

High levels of transcriptional activity occur within the epididymis.^32^ Resveratrol improves sperm DNA quality and reproductive capacity in type 1 diabetes.^60^ The highest levels of Glu‐TRH, which is a sperm capacitation factor,^32^ occur within the epididymis and were significantly increased by acute and withdrawal treatments with PT (Table 3).

The tissue in male rats with the highest levels of TRH and TRH‐like peptides is the prostate. The TRH levels are subject to a 12‐fold variation during the 24‐h photoperiod with highest level during the diurnal period.^32^ Because rats are nocturnal while humans are most active during the day, this may explain the approximately 10‐fold higher levels of rat TRH immunoreactivity (TRH‐IR) in daytime compared to humans.^32^ The highly significant increases in Glu‐TRH levels in response to PT treatments (Table 3 and Figure 3) are of particular interest because prostate cancer in particular, and other cancers, in general, have been found to be associated with nerves^18^ which are the main source of these peptides. They are co‐secreted with glutamate and other neurotoxic stress‐related neurotransmitters.^32^ Prostatic fluid contains TRH and other TRH‐like peptides and appears to be secreted by epithelial cells.^32^

Oral PT has been reported to improve stress‐related behaviours, neuroinflammation and hormonal changes in a mouse stress model.^6^ Acute and chronic PT treatment decreased adrenal TRH, Tyr‐TRH, Leu‐TRH and Phe‐TRH levels in the adrenals (Table 3) consistent with increased release of these antidepressant peptides.^32^, ^44^

Chronic treatment with PT reduced serum glucose (Table 1) and increased pancreatic Val‐TRH and decreased Tyr‐TRH levels (Table 3). Resveratrol increases levels of SIRT1 which attenuates serum glucose in diabetic rats by increasing insulin production and insulin sensitivity.^61^

Pterostilbene and resveratrol strongly modulate ghrelin and leptin levels while these metabolism‐ and obesity‐related proteins profoundly alter the expression of TRH and TRH‐like peptides.^32^ Alterations in the gut microbiome in response to modern lifestyles, and ageing^34^ suggest the examination of the extent of ghrelin and leptin mediation of PT modulation of TRH and TRH‐like peptide expression is warranted.

## 5 CONCLUSIONS

Acute, chronic and withdrawal treatment with oral PT has significant effects on the expression of TRH and TRH‐like peptides throughout the brain and peripheral tissues of male rats. These effects are consistent with these tripeptides playing a significant role in the antidepressant, anti‐ataxic, anti‐autistic, neuroprotective, antihypertensive, anti‐ageing, anxiolytic and reproductive effects of this resveratrol analog which readily crosses the blood–brain barrier and thereby enhances its bioavailability.

## AUTHOR CONTRIBUTIONS

**Albert Eugene Pekary:** Conceptualization (equal); data curation (equal); formal analysis (equal); funding acquisition (equal); investigation (equal); methodology (equal); project administration (equal); resources (equal); software (equal); supervision (equal); validation (equal); visualization (equal); writing – original draft (equal); writing – review and editing (equal). **Albert Sattin:** Investigation (equal); methodology (equal); writing – review and editing (equal).

## CONFLICT OF INTEREST

None.

## Data Availability

All statistically summarized data are included in this published article. Primary data are available from AEP upon reasonable request.
